# Changes in iron metabolism during prolonged repeated walking exercise in middle-aged men and women

**DOI:** 10.1007/s00421-018-3961-5

**Published:** 2018-08-23

**Authors:** Rieneke Terink, D. ten Haaf, C. W. G. Bongers, M. G. J. Balvers, R. F. Witkamp, M. Mensink, T. M. H. Eijsvogels, J. M. T. Klein Gunnewiek, M. T. E. Hopman

**Affiliations:** 1 Division of Human Nutrition, Wageningen University (WU), Stippeneng 4, building 124, 6708 WE Wageningen, The Netherlands; 2 Department of Physiology, Radboud University Nijmegen, Nijmegen, The Netherlands; 3 Clinical Chemistry and Haematology Laboratory, Gelderse Vallei Hospital (ZGV), Ede, The Netherlands

**Keywords:** Hb, Fe, Hp, Repetitive exercise

## Abstract

**Purpose:**

The aim of the present study was to assess the effect of prolonged and repeated exercise on iron metabolism in middle-aged adults and to compare differences between sexes.

**Methods:**

50 male (58.9 ± 9.9 year) and 48 female (50.9 ± 11.2 year) individuals were monitored on 4 consecutive days at which they walked on average 8 h and 44 min per day at a self-determined pace. Blood samples were collected 1 or 2 days prior to the start of the exercise (baseline) and every day immediately post-exercise. Samples were analysed for iron, ferritin, haemoglobin, and haptoglobin concentrations.

**Results:**

Plasma iron decreased across days, while ferritin increased across days (both *p* < 0.001). Haptoglobin showed a decrease (*p* < 0.001) after the first day and increased over subsequent days (*p* < 0.001). Haemoglobin did not change after the first day, but increased during subsequent days (*p* < 0.05). At baseline, 8% of the participants had iron concentrations below minimum reference value (10 µmol/L), this increased to 43% at day 4. There was an interaction between sex and exercise days on iron (*p* = 0.028), ferritin (*p* < 0.001) and haemoglobin levels (*p* = 0.004), but not on haptoglobin levels.

**Conclusion:**

This study showed decreases in iron, increases in ferritin, a decrease followed by increases in haptoglobin and no change followed by increases in haemoglobin. This is most likely explained by (foot strike) haemolysis, inflammation, and sweat and urine losses. These processes resulted in iron levels below minimum reference value in a large number of our participants.

## Introduction

Iron is involved in several processes related to exercise (Buratti et al. 2015; Peeling et al. 2008; Lukaski 2004). Iron deficiency can lead to acute and chronic health problems, and to underperformance (Lukaski 2004; Reinke et al. 2012). Vice versa, exercise can affect iron status via various mechanisms, including exercise-induced inflammation (Peeling et al. 2008, 2009a), foot strike haemolysis (Peeling et al. 2009a; Telford et al. 2003; Tan et al. 2012), and losses through sweat or urine (DeRuisseau et al. 2002; McInnis et al. 1998).

Exercise-induced inflammation may lead to increased ferritin and hepcidin plasma levels (Peeling et al. 2009a; Banzet et al. 2012), which are both categorized as acute phase proteins. The rise in hepcidin levels causes a reduced influx of iron in the circulation through increased degradation of ferroportin (Nemeth et al. 2004), which might result in iron deficiency in the long run (Ganz and Nemeth 2006; Latunde-Dada 2013). Next to this, certain types of exercise can lead to foot strike haemolysis. This causes destruction of red blood cells, resulting in the release of haemoglobin and as a consequence a decrease in haptoglobin. Haptoglobin binds free haemoglobin resulting in lower haptoglobin levels due to clearance of this complex. Foot strike haemolysis has, for example, been reported following 1 h of running exercise (Telford et al. 2003), repeated exercise (10 times 1 km running) (Peeling et al. 2009a) and football-specific training (Tan et al. 2012). Sweating is another cause of iron loss during exercise (DeRuisseau et al. 2002). And finally, blood loss through urine, i.e., hematuria, and blood loss through the breakdown of muscle, i.e., myoglobinuria, can occur after exercise as well, for example after a 400 m sprint at maximal effort (McInnis et al. 1998). The vast majority of studies on exercise-induced changes in iron status have focused on well-trained athletes, during short and relatively intensive forms of exercise. However, far less is known about the effects of repeated moderate intensity endurance exercise in other populations, including middle-aged recreational sportsmen/women.

Therefore, the aim of the present study was to assess the effect of prolonged and repeated walking exercise on iron parameters in middle-aged men and women.

We hypothesised that iron, ferritin, haemoglobin, and haptoglobin levels would change during prolonged and repeated walking exercise. We expected ferritin to increase and iron to decrease. In addition, we expected a decrease in haptoglobin after the first day and an increase at consecutive days, due to (foot strike) haemolysis and inflammation, respectively. Finally, we expected an increase in haemoglobin after the first day and a decrease at consecutive days, due to haemoconcentration and haemodilution, respectively.

## Materials and methods

### Study population

We selected 50 male and 50 female walkers who participated in the 2015 edition of the Nijmegen Four Days Marches, a large annual walking event taking place in the Netherlands (http://www.4daagse.nl/en/). Exclusion criteria were known diabetes and/or renal dysfunction. The study was approved by the Medical Ethical Committee of the Radboud university medical center (CMO registration number: 2007/148), and all participants gave written informed consent prior to participation. This study was conducted in accordance with the Declaration of Helsinki.

### Study procedure

Measurements were performed before the start of the event (‘baseline’), and at the four consecutive walking days. Baseline measurements, including recording participant characteristics, a blood sample, and questionnaires, were performed in our field laboratory at the event location 1 or 2 days prior to the first walking day, between 09:30 AM and 04:00 PM.

Every walking day, immediately before the start of the march, participants’ body weight was determined. Thereafter, participants walked 30, 40 or 50 km, at a self-determined pace, starting between 4:00 and 8:00 AM. Every day, participants registered their fluid intake using a diary. Directly after finishing, post-exercise body weight was determined, a blood sample was taken and questionnaires were completed. Heart rate was measured every 5 km and at the finish during the first walking day using a 2-channel ECG chest band system (Polar Electro Oy, Kempele, Finland). Mean heart rate during exercise was calculated as the average heart rate, excluding the values derived directly before the start and after the finish. Heart rate (HR) was used to estimate exercise intensity: Intensity = (Measured HR/estimated maximal HR) * 100%, with estimated max HR = 208 − (0.7 * Age) (Tanaka et al. 2001).

### Baseline measurements

At baseline, body weight (Seca 888 scale, Hamburg, Germany) and body height were determined and body mass index (BMI) was calculated. Thereafter, resting heart rate was measured using an automated sphygmomanometer (M5-1 intellisense, Omron Healthcare, Hoofddorp, The Netherlands) after 5 min supine rest.

### Blood samples

Participants were seated for 5 min after which a venous blood sample was taken from the cephalic vein. Blood was collected in a 4 ml Lithium Heparin (LH) gel vacutainer (Becton–Dickinson, New Jersey, America). The vacutainer was centrifuged at 3000*G* (3755 rpm) for 8 min at 22° and plasma was stored at − 80 °C. Samples were analysed for their iron, ferritin and haptoglobin concentrations in October 2015 (Siemens Dimension Vista 1500, Siemens Healthcare, Erlangen, Germany).

An additional blood sample was collected in a 2 ml LH vacutainer (Becton–Dickinson, New Jersey, USA) and used for direct analyses of plasma haemoglobin and haematocrit concentrations (Rapidpoint 400, Siemens Healthcare Diagnostics Inc., Tarrytown, New York, USA). Relative changes in plasma volume were calculated from blood haematocrit and haemoglobin concentrations using Dill and Costill’s equation (Dill and Costill 1974). Iron, ferritin, haptoglobin and haemoglobin levels were corrected for plasma volume changes.

### Urine samples

Urine samples were collected after exercise and with the use of a urinary dipstick (Clinitek Status® analyzer, Siemens Healthcare diagnostics Inc., Tarrytown, New York) tested for the presence of erythrocytes, haemoglobin and/or myoglobin.

### Questionnaires

All participants completed a questionnaire about the use of supplements to check whether iron concentrations could have been influenced by iron supplement use and for female participants we collected data about their menstrual status. All participants filled out a food frequency questionnaire (FFQ) before the start of the event to estimate dietary intake.

### Statistical analysis

Statistical analyses were performed using Statistical Package for Social Sciences 22.0 (IBM SPSS version 22.0, Armonk, New York, USA), with the level of significance set at *p* < 0.05. Data were presented as mean ± SD unless indicated otherwise. A post hoc power analysis was conducted in GPower (version 3.0.10, Dusseldorf, Germany), revealing a power > 0.95 for all four parameters.

The Kolmogorov–Smirnov test was used to examine the normality of the data distribution. Participant and exercise characteristics were analysed with an independent Student’s *t* test to examine sex differences. A backward linear regression was used to analyse which variables contributed significantly to the observed changes in iron, ferritin, haptoglobin and haemoglobin.

Normally distributed data were analysed using ANOVA repeated-measures for the effect of consecutive exercise days, with a Bonferroni post hoc correction. A two-way repeated-measures ANOVA was applied to analyse sex differences (sex × days). Non-parametric tests were used for analysing not-normally distributed data. This included a Friedman’s test, to evaluate the effect of consecutive exercise days on iron parameters. When significant differences were found, a Wilcoxon signed-rank test was used to determine which consecutive days significantly differed from one another.

## Results

### Characteristics of the study population

Two female participants did not finish the first day, due to back problems, and were excluded from further analysis. The characteristics of the remaining 98 participants who completed all 4 days are shown in Table 1. Men had a significantly higher age, height, weight, BMI, macronutrient intake, fibre intake, iron intake (total, haem and non-haem), vitamin B6 and B12 intake (*p* < 0.05) compared to women. There were no differences in resting heart rate, average training distance in the year prior to the marches and folic acid intake between men and women (*p* > 0.05).

**Table 1:** Participants characteristics

	Men (*n* = 50)	Women (*n* = 48)	*p* value
Characteristics			
Age (years)	58.9 ± 9.9	50.9 ± 11.2	< 0.001
Body composition			
Height (cm)	180 ± 6	167 ± 6	< 0.001
Weight (kg)	88.9 ± 13.3	65.6 ± 8.1	< 0.001
BMI (kg/m^2^)	27.3 ± 3.3	23.5 ± 3.0	< 0.001
Cardio characteristics			
Resting heart rate (bpm)	63 ± 9.9	64 ± 6.9	0.758
Dietary intake			
Energy intake (*103 kJ)	10.7 ± 2.8	8.0 ± 2.5	< 0.001
Total protein (g)	95 ± 25	75 ± 25	< 0.001
Total fat (g)	104 ± 35	75 ± 27	< 0.001
Total carbohydrates (g)	268 ± 78	204 ± 72	< 0.001
Fibre (g)	27 ± 8	22 ± 8	0.005
Iron intake (via diet)			
Total iron (mg)	13 ± 3	11 ± 3	< 0.001
Haem iron (mg)	1.2 ± 0. 6	0.9 ± 0.5	0.006
Non-haem iron (mg)	12 ± 3	10 ± 3	0.001
Vitamin B6 (mg)	2.1 ± 0.8	1.8 ± 0.7	0.022
Vitamin B12 (µg)	6.0 ± 2.6	4.7 ± 2.4	0.014
Folic acid total (µg)	289 ± 78	257 ± 86	0.059
Walking characteristics			
Training distance (km)	812 ± 1072	753 ± 921	0.770
30 km/day	*n* = 17	*n* = 5	
40 km/day	*n* = 20	*n* = 39	
50 km/day	*n* = 13	*n* = 4	

Means ± SD are shown. *BMI* Body mass index; dietary intake values are estimated with a FFQ questionnaire; Training distance: specific walking distance in the year prior to the 4 Days Marches; *p* value refers to an unpaired Student’s *t* test between men and women

Twenty of our 48 female participants were post-menopausal, 5 women were not sure. We did not collect data about the stage of menstruation for those female participants who were pre-menopausal. There were no significant differences in iron, ferritin, haemoglobin and haptoglobin levels between post-menopausal women and pre-menopausal women at baseline and during walking days. Therefore, we treat them as one group for all following data.

### Nutritional iron intake and use of supplements

Supplements of any kind were used by 33.6% (*n* = 37) of our participants. Iron supplements were used by only 1 female participant. Total iron intake for men was significantly higher (13.2 ± 3.0 mg per day) compared to iron intake for women (10.9 ± 3.2 mg per day) (*p* < 0.001) (RDA is 8 mg/day and 18 mg/day for men and women, respectively (Trumbo et al. 2001)) (Table 1). The dietary variables did not significantly contribute to the observed changes in iron, ferritin, haptoglobin or haemoglobin.

### Exercise characteristics

Walking exercise intensity assessed as percentage of estimated maximal heart rate, for all participants was 67 ± 9%, with an average heart rate of 114 ± 17 bpm (Table 2). Exercise intensity, was not different between sexes (*p* = 0.679).

**Table 2:** Exercise characteristics presented for men and women

	Day 1	Day 2	Day 3	Day 4	Asymp. sign.
Walking					
Exercise duration (hours:minutes)					
Men	7:52 ± 2:32	8:32 ± 1:31	8:23 ± 1:45	8:51 ± 2:03	0.001
Women	8:43 ± 1:04	9:05 ± 1:16	8:41 ± 2:15	9:45 ± 1:32	< 0.001
*p* value	0.032	0.054	0.459	0.017	
Speed (km/h)					
Men	4.8 ± 0.8	4.6 ± 0.8	4.7 ± 0.7	4.5 ± 0.8	0.003
Women	4.6 ± 0.6	4.4 ± 0.7	4.5 ± 0.6	4.2 ± 0.6	< 0.001
*p* value	0.235	0.186	0.046	0.009	
Fluid intake (L)					
Men	4.4 ± 1.6	4.4 ± 1.5	4.3 ± 1.3	2.8 ± 1.1	< 0.001
Women	4.1 ± 1.7	3.8 ± 1.5	3.6 ± 1.6	2.6 ± 1.0	< 0.001
*p* value	0.367	0.034	0.015	0.397	
Physical parameters					
Weight difference (kg)					
Men	− 1.4 ± 1.0	− 0.8 ± 0.7	− 0.5 ± 0.6	− 0.9 ± 0.8	< 0.001
Women	− 0.3 ± 0.7	− 0.3 ± 0.5	− 0.3 ± 0.5	− 0.5 ± 0.5	0.009
*p* value	0.000	0.000	0.158	0.016	
Plasma volume change from baseline (%)					
Men	− 4.29 ± 12.21	− 1.93 ± 8.34	3.62 ± 10.37	6.05 ± 8.98	< 0.001
Women	0.001 ± 5.91	3.40 ± 6.25	7.82 ± 7.45	8.67 ± 7.08	< 0.001
*p* value	0.030	0.001	0.024	0.113	
Mean heart rate (bpm)					
Men	113 ± 18				
Women	115 ± 15				
*p* value	0.512				
Max heart rate (bpm)					
Men	124 ± 27				
Women	129 ± 18				
*p* value	0.260				
Exercise intensity (%HRmax)					
Men	67 ± 11				
Women	67 ± 8				
*p* value	0.679				

Values are mean values for the 4 walking days. *p* values refers to an unpaired Student’s *t* test between men and women. Asymp. Sign *p* values refer to an Intra-group Friedman ANOVA test for the effect of days. Weight difference is calculated as post-exercise − pre-exercise, a negative value means weight loss. Plasma volume change is calculated as day # − baseline, plasma volume is calculated with Dill and Costill calculation (1974)

Male participants walked 30 km (*n* = 17), 40 km (*n* = 20) and 50 km (*n* = 13). Female participants walked the same distances (*n* = 5, *n* = 39 and *n* = 4, respectively). Exercise duration and speed were different between men and women, as well as plasma volume changes (Table 2). Exercise intensity significantly contributed to the observed change in ferritin. Exercise intensity and duration did not significantly contribute to the observed changes in iron, haptoglobin or haemoglobin.

### Laboratory parameters

#### Iron

**Fig. 1:**
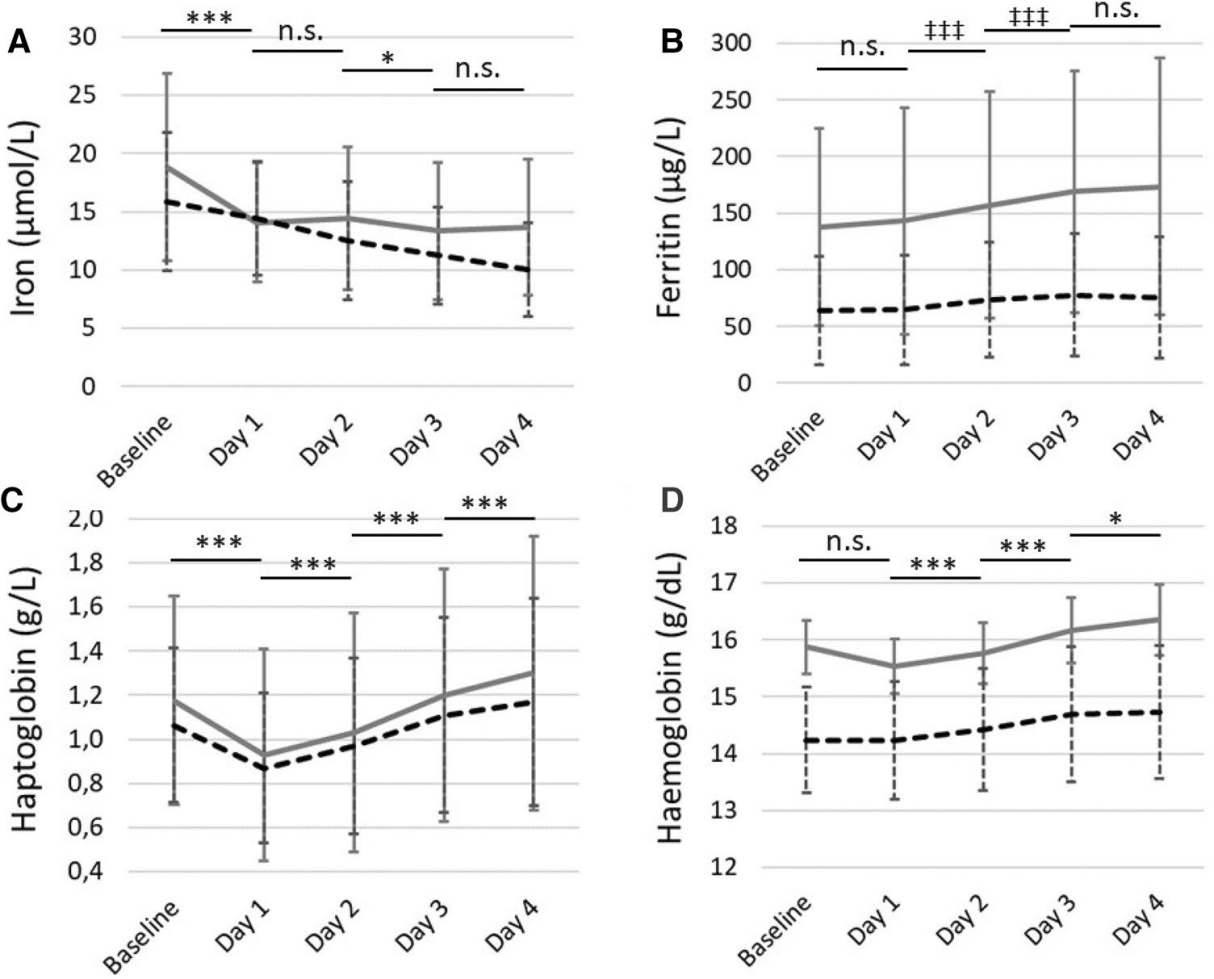
Iron, ferritin, haptoglobin and haemoglobin levels for men (grey continuous line) and women (black dotted line). Means ± SD are shown. Significant differences between days for all participants together are presented with horizontal lines, with *p < 0.05, **p < 0.01, and ***p < 0.001 for repeated measures ANOVA, with ^‡^*p* < 0.05, ^‡‡^*p* < 0.01, and ^‡‡‡^*p* < 0.001 for Friedman test

Baseline iron concentrations were significantly higher in men compared to women (Table 3), while 11% of the study population had a plasma iron concentration below the minimum reference value of 10 µmol/L. A significant decrease in iron concentrations was observed across days (Fig. 1a) (*p* < 0.001), with a larger decrease in men compared to women after the first walking day (Interaction = 0.028). Iron concentrations for men fluctuated, with a significant decrease after the first day of exercise, and no significant changes after that, while women showed a continuous decrease over days, although this was not significant between days. The percentage of participants below reference value increased significantly from 1 to 28% in men and from 10 to 52% in women, from baseline to the last exercise day.

**Table 3:** Mean laboratory parameter concentrations for men and women separately, at baseline, and day 1 until day 4

	Baseline	Day 1	Day 2	Day 3	Day 4	Asymp. sign.
Iron (µmol/L)						
Men	18.83 ± 8.07	14.07 ± 5.11	14.45 ± 6.14	13.36 ± 5.89	13.67 ± 5.82	< 0.001
Women	15.86 ± 5.94	14.46 ± 4.87	12.51 ± 5.04	11.22 ± 4.13	10.04 ± 4.05	< 0.001
*p* value	0.045	0.708	0.092	0.041	0.001	
Ferritin (µg/L)						
Men	137.72 ± 87.03	143.16 ± 99.86	157.34 ± 100.25	169.00 ± 107.02	173.60 ± 113.51	< 0.001
Women	64 ± 48.23	64.92 ± 48.39	73.66 ± 50.83	77.80 ± 54.04	75.55 ± 54.00	< 0.001
*p* value	0.000	0.000	0.000	0.000	0.000	
Haptoglobin (g/L)						
Men	1.175 ± 0.472	0.93 ± 0.48	1.03 ± 0.54	1.20 ± 0.57	1.30 ± 0.62	< 0.001
Women	1.064 ± 0.35	0.87 ± 0.34	0.97 ± 0.40	1.11 ± 0.44	1.17 ± 0.47	< 0.001
*p* value	0.197	0.516	0.575	0.378	0.237	
Haemoglobin (g/dL)						
Men	15.874 ± 1.139	15.53 ± 1.51	15.76 ± 1.48	16.17 ± 1.65	16.35 ± 1.63	0.022
Women	14.24 ± 0.933	14.23 ± 1.04	14.43 ± 1.07	14.69 ± 1.19	14.73 ± 1.16	< 0.001
*p* value	0.000	0.000	0.000	0.000	0.000	

*n* = 98. Values are mean ± SD for baseline and the four walking days. Values are corrected for plasma volume changes. *p* values refer to an unpaired Students *t* test between male and female participants. Asymp. Sign *p* values refer to an Intra-group Friedman ANOVA test for the effect of days for non-parametric data and repeated-measures ANOVA for the effect of days on parametric data

#### Ferritin

Ferritin concentrations were significantly higher in men compared to women (Table 3). A significant increase in ferritin concentrations was observed across days (Fig. 1b) (*p* < 0.001), with varying changes in men and women across days (Interaction < 0.001). Ferritin concentrations increased all walking days in men, while it decreased on the final walking day in women.

#### Haptoglobin

Haptoglobin concentrations at baseline were not significantly different between men and women (Table 3). A significant decrease in haptoglobin concentrations was observed after the first day of exercise, followed by an increase over subsequent days (Fig. 1c) (*p* < 0.001), with no differences in change between men and women (Interaction = 0.249).

#### Haemoglobin

Haemoglobin concentrations at baseline were significantly higher in men compared to women (Table 3). A significant change in haemoglobin concentrations was observed across days (Fig. 1d) (*p* < 0.001), with no change after the first day of walking, and increases over subsequent days. Increases in men were larger compared to women (Interaction = 0.004).

#### Iron losses via urine

The number of participants with iron loss in urine was 26% at baseline, which increased during the 4 walking days (Table 4). At the final walking day, 54% of the participants had iron loss through urine.

**Table 4:** Iron loss via urine

Parameter	Baseline	Day 1	Day 2	Day 3	Day 4
Negative, *n* (%)	72 (74)	50 (51)	54 (55)	52 (53)	45 (46)
Positive, *n* (%)	26 (26)	48 (49)	44 (45)	46 (47)	53 (54)

## Discussion

The changes in biochemical parameters found in our study population are consistent with the occurrence of an inflammatory response, haemolysis and iron losses during this prolonged and repeated exercise. We found a cumulative decrease in iron, an increase in ferritin, a decrease in haptoglobin after 1 day of walking followed by an increase on subsequent days and an increase in haemoglobin from walking day 2 until 4. The implications of these results are discussed below.

### Iron

Our finding that iron levels decreased after 1 day of exercise are in line with results reported after a marathon (Roecker et al. 2005) and a 100 km ultramarathon (Chiu et al. 2015). On the other hand, opposite findings with increased iron levels after a triathlon (Peeling et al. 2014), and a marathon (Buchman et al. 1998) have been reported as well. These increased iron levels are probably caused by decreased plasma volumes. In contrast to previous studies, we corrected our results for changes in plasma volumes, and observed a decrease in iron levels after the first day.

Changes in iron levels during this type of exercise are probably related to inflammation and loss via urine. In a previous study, using the same study population, we showed that inflammation was evident by elevated cytokine levels after the first day of walking (not yet published work). This included IL-6 which has been shown to enhance hepcidin levels (Peeling et al. 2009b). Hepcidin is also known as acute phase protein, reducing iron influx into the circulation by binding to ferroportin and inducing its internalization (Nemeth et al. 2004). This could partly explain our observed decrease in iron.

In addition, we found an increase in iron loss through urine, which might also contribute to decreased iron levels. We could not distinguish between iron losses through urine by haemoglobinuria and/or myoglobinuria since the dipsticks used gave a positive test result for both. Nevertheless, we showed that this type of exercise caused iron losses through urine.

We observed a further decrease in iron after repeated days of walking exercise, which was in contrast to previous studies (Bourque et al. 1997; Wilkinson et al. 2002). These studies were of longer duration (weeks), while we assessed the effect of 4 days of exercise on iron levels.

The continuous decrease in iron may be related to urinary iron loss, which continued over the 4 days, while inflammation may still play a role, as mentioned above.

### Ferritin

We found no significant increase in ferritin after the first day of exercise, which was in contrast to results reported after a 100 km ultramarathon (Chiu et al. 2015), a triathlon race (Rocker et al. 2002) and a 2000 m rowing ergometer test (Skarpanska-Stejnborn et al. 2015). Exercise intensity differed a lot between these studies and our walking exercise. Previous studies were all-out efforts, while our walking exercise was at a moderate intensity. This could explain the observed differences.

We found a significant increase in ferritin after the second and third day of walking exercise, which was in line with previous studies (Pattini et al. 1990; McClung et al. 2013). Ferritin also belongs to the acute phase proteins, showing increased levels during inflammation (Peeling et al. 2008), which was found to occur during this walking exercise (unpublished work). In addition, ferritin synthesis is increased in response to oxidative stress (Orino et al. 2001), and ferritin levels increase after leakage from damaged cells (Kell and Pretorius 2014), like peripheral (and intestinal) cells (Kell 2009). Both mechanisms possibly occurred during this event. Previous studies have shown that, for example, gastrointestinal blood loss may occur during exercise (Sullivan 1986).

### Haptoglobin and haemoglobin

We found a decrease in haptoglobin after the first day of walking, which was in line with previous studies concerning running exercise (Peeling et al. 2009a; Telford et al. 2003). Haptoglobin decrease has been linked to haemolysis. Haemoglobin from damaged red blood cells is released into plasma, causing oxidative stress. This is limited by haptoglobin, which forms a complex with haemoglobin (Schaer et al. 2014). The haptoglobin–haemoglobin complex is cleared from the circulation by binding to CD163 haemoglobin scavenger receptors on the surface of macrophages. After that, the complex is taken up by endocytosis in the liver (Schaer et al. 2014).

Interestingly, we found larger decreases in plasma haptoglobin compared to previous studies (Peeling et al. 2009a; Telford et al. 2003). Probably, the long distance and duration of the exercise in our study (~ 9 h) caused a higher degree of foot strike haemolysis compared to these shorter exercises (10 km or 1 h, respectively) (Peeling et al. 2009a; Telford et al. 2003). Our measured decrease in haptoglobin levels was comparable with depressed levels reported directly after marathon running (Egan et al. 1987) and a 160 km ultramarathon (Taylor et al. 1987).

We found increased levels of haptoglobin after repeated walking days. This can be explained by the ongoing inflammation causing haptoglobin, which is a positive acute phase protein (Mihara et al. 2012), to increase. To our knowledge, studies reporting changes in haptoglobin after repeated exercise are scarce. One study showed an opposite result, with a cumulative effect of consecutive running sessions on haemolysis shown by a greater decrease in haptoglobin during a second running session (Peeling et al. 2009a). In that specific study, inflammation was not affected by the second running session, resulting in further decreases in haptoglobin (Peeling et al. 2009a).

In contrast to previous studies on prolonged walking exercise (Gilligan and Altschule 1950; Hornbostel 1970) we found no significant change in haemoglobin after the first day of walking exercise and significant increases on subsequent days. Decreases in haemoglobin levels are often mistaken for anaemia, while most of these changes are probably caused by changes in plasma volume. An acute bout of exercise mostly results in temporary haemoconcentration (Singh and Sirisinghe 1999), while repeated exercise results in expansion of plasma volume (Convertino 2007).

Indeed, our data prior to correction for plasma volume changes showed an increase in haemoglobin after the first day of walking and a decrease after subsequent days of walking. This is in line with our observed plasma volume changes, which showed haemoconcentration after the first day of walking exercise and haemodilution after subsequent days of walking exercise. Therefore, it is important to take plasma volume changes into account during these types of exercise studies.

### Differences between men and women

We found comparable haptoglobin changes between men and women, which is in line with results from a marathon race (Weight et al. 1991), suggesting that foot strike haemolysis occurred in both groups. On the other hand, ferritin, haemoglobin and iron changes were different between men and women, which have not been reported in detail before. The magnitude of the increases and decreases differed between men and women; however, the overall trend in all parameters was roughly comparable. Therefore, we suggest that mechanisms, like inflammation, haemolysis and blood losses were present in both male and female walkers. Why the magnitude of changes differs between men and women is unknown, but it could be related to differences in exercise intensity, sweat rate and body composition between men and women.

Interestingly, iron levels and changes in our middle aged women were comparable with female athletes, who are studied more often in relation to iron and exercise (DellaValle 2013; Sandstrom et al. 2012; Dellavalle and Haas 2012), as they are at risk for an iron deficiency with and without anaemia (McClung 2012). This is mainly caused by iron losses during menstruation. A large number of our female participants were already post-menopausal. Therefore, one might expect smaller differences in the iron levels between our male and female participants. However, that was not the case, suggesting that (older) female adults participating in sports should also be aware of this decrease in iron levels during exercise.

### Strengths and limitations

The present study had some limitations which are mainly due to practical reasons. First, we did not include a pre-exercise blood withdrawal in the early morning. Instead we had blood drawings at baseline.

The strength of the present study was the large number of participants in which we were able to study the effect of repeated exercise. Furthermore, our study population, characterised as generally healthy, regularly exercising middle-aged persons is quite unique and apparently underrepresented in the field of exercise physiology.

## Conclusion

The present study shows decreases in iron, increases in ferritin, a decrease followed by increases in haptoglobin and no change followed by increases in haemoglobin. These changes are most likely the result of (foot strike) haemolysis, inflammation and sweat and urine losses occurring during this type of exercise. These processes during exercise resulted in iron levels below minimum reference value in a large number of our participants.

## Abbreviations

ANOVA: Analysis of variance
BMI: Body mass index
FFQ: Food frequency questionnaire
HR: Heart rate
LH: Lithium heparine
Rpm: Revolutions per minute
